# Inhibition of macrophage infectivity potentiator in *Burkholderia pseudomallei* suppresses pro-inflammatory responses in murine macrophages

**DOI:** 10.3389/fcimb.2024.1353682

**Published:** 2024-03-25

**Authors:** Jua Iwasaki, Nicole M. Bzdyl, Dion J. M. Lin-Sullivan, Nicolas J. Scheuplein, Maria Emilia Dueñas, Emma de Jong, Nicholas J. Harmer, Ulrike Holzgrabe, Mitali Sarkar-Tyson

**Affiliations:** ^1^Marshall Centre for Infectious Disease Research and Training, School of Biomedical Sciences, The University of Western Australia, Perth, WA, Australia; ^2^Wesfarmers Centre of Vaccines and Infectious Diseases, Telethon Kids Institute, University of Western Australia, Nedlands, WA, Australia; ^3^Centre for Child Health Research, University of Western Australia, Perth, WA, Australia; ^4^Institute of Pharmacy and Food Chemistry, University of Würzburg, Würzburg, Germany; ^5^Medical School, The University of Western Australia, Perth, WA, Australia; ^6^Department of Biosciences, University of Exeter, Geoffrey Pope Building, Exeter, United Kingdom; ^7^Living Systems Institute, Faculty of Health and Life Sciences, University of Exeter, Exeter, United Kingdom

**Keywords:** *Burkholderia pseudomallei*, macrophage infectivity potentiator, small-molecule inhibitor, immune response, RNA sequencing

## Abstract

**Introduction:**

Melioidosis, caused by the Gram-negative bacterium *Burkholderia pseudomallei*, is a disease endemic in many tropical countries globally. Clinical presentation is highly variable, ranging from asymptomatic to fatal septicemia, and thus the outcome of infection can depend on the host immune responses. The aims of this study were to firstly, characterize the macrophage immune response to *B. pseudomallei* and secondly, to determine whether the immune response was modified in the presence of novel inhibitors targeting the virulence factor, the macrophage infectivity potentiator (Mip) protein. We hypothesized that inhibition of Mip in *B. pseudomallei* would disarm the bacteria and result in a host beneficial immune response.

**Methods:**

Murine macrophage J774A.1 cells were infected with *B. pseudomallei* K96243 in the presence of small-molecule inhibitors targeting the Mip protein. RNA-sequencing was performed on infected cells four hours post-infection. Secreted cytokines and lactose dehydrogenase were measured in cell culture supernatants 24 hours post-infection. Viable, intracellular *B. pseudomallei* in macrophages were also enumerated 24 hours post-infection.

**Results:**

Global transcriptional profiling of macrophages infected with *B. pseudomallei* by RNA-seq demonstrated upregulation of immune-associated genes, in particular a significant enrichment of genes in the TNF signaling pathway. Treatment of *B. pseudomallei*-infected macrophages with the Mip inhibitor, AN_CH_37 resulted in a 5.3-fold reduction of *il1b* when compared to cells treated with DMSO, which the inhibitors were solubilized in. A statistically significant reduction in IL-1β levels in culture supernatants was seen 24 hours post-infection with AN_CH_37, as well as other pro-inflammatory cytokines, namely IL-6 and TNF-α. Treatment with AN_CH_37 also reduced the survival of *B. pseudomallei* in macrophages after 24 hours which was accompanied by a significant reduction in *B. pseudomallei*-induced cytotoxicity as determined by lactate dehydrogenase release.

**Discussion:**

These data highlight the potential to utilize Mip inhibitors in reducing potentially harmful pro-inflammatory responses resulting from *B. pseudomallei* infection in macrophages. This could be of significance since overstimulation of pro-inflammatory responses can result in immunopathology, tissue damage and septic shock.

## 1 Introduction

Melioidosis, caused by the Gram-negative bacterium *Burkholderia pseudomallei*, is a disease endemic in Southeast Asia and northern Australia, with increasing cases across the Pacific, South Asia, Africa and the Americas (Limmathurotsakul et al., 2016; Currie et al, 2023; Meumann et al., 2024). Infections can be caused through subcutaneous inoculation, inhalation, or ingestion of the bacterium. Several features of melioidosis suggest that host immune mechanisms play an important role in the outcome of infection. This includes a broad spectrum of clinical manifestations, from asymptomatic to chronic illness to fatal acute septicemia (Currie et al, 2010; Meumann et al., 2024). The host’s immune response to acute infection, which constitutes approximately 85% of melioidosis cases, can control bacterial growth and eradicate infection in most healthy individuals (Currie et al, 2010; Wiersinga et al., 2018). However, multiple risk factors for developing melioidosis have been identified and in patients with comorbidities, such as diabetes mellitus, there is a high association with susceptibility to infection and risk of critical illness (Cheng and Currie, 2005), further highlighting the importance of host immunity in dictating the disease trajectory.

In addition to host factors, the ability of *B. pseudomallei* to efficiently evade or manipulate the immune response of the host plays a key role in survival and establishment of infection. *B. pseudomallei* has been shown to evade macrophage-mediated clearance and contribute to persistence within macrophages through interference of inducible nitric oxide (iNOS) expression, an enzyme that catalyzes nitric oxide (NO) production and is important for intracellular bacterial killing (Utaisincharoen et al., 2001). The possible role of the O-antigenic polysaccharide moiety of lipopolysaccharide in this mechanism has been suggested (Arjcharoen et al., 2007). Additionally, the type III secretion system cluster 3 (TTSS3) effector protein BopA has been identified as a virulence factor, playing an important role in escaping from phagosomes and evading autophagy in murine macrophages (Cullinane et al., 2008; Gong et al., 2011). Finally, *B. pseudomallei* actively inhibits NF-κB and type I IFN pathway activation, thereby downregulating host inflammatory responses. This modulation of the innate immune response was attributed to TssM, a virulence factor with deubiquitinase activity (Tan et al., 2010).

The macrophage infectivity potentiator (Mip) protein, belonging to the FK506-binding protein (FKBP) family of the immunophilin superfamily, is a novel virulence factor that has pleiotropic effects in *B. pseudomallei* infection including resistance to host killing mechanisms (Norville et al., 2011a). FK506 and rapamycin can potently inhibit the enzymatic function of Mip, however both are unsuitable for use as *in vivo* antimicrobial therapy due to their immunosuppressive effects (Wang et al, 2022). To overcome this, the pipecolic acid domain of rapamycin was used to generate non-immunosuppressive small-molecule compounds that could inhibit the characteristic peptidyl-prolyl *cis*-*trans* isomerase activity (PPIase) activity of the Mip protein (Juli et al., 2011; Begley et al., 2014; Seufert et al., 2016; Scheuplein et al., 2023). We recently demonstrated that these compounds could inhibit the PPIase activity of Mip in *B. pseudomallei* and reduce bacteria-induced cytotoxicity in murine macrophages (Iwasaki et al., 2022; Scheuplein et al., 2023). In the present study, we expand on these findings to characterize the macrophage immune response to *B. pseudomallei* in the presence of two lead Mip inhibitors using RNA sequencing and immunoassays. We hypothesize that disarming *B. pseudomallei* using Mip inhibitors will enable control of the bacterium in macrophages and reduce the development of clinically relevant host damage and inflammation.

## 2 Materials and methods

### 2.1 Inhibitor synthesis

Mip inhibitors, SF235 (Seufert et al., 2016) and AN_CH_37 (Scheuplein et al., 2023) were synthesized as described previously (Australian Patent Application No. PCT/AU2023/050201). Inhibitors were solubilized in DMSO.

### 2.2 Peptidyl-prolyl *cis/trans* isomerase assay

Recombinant *B. pseudomallei* Mip (*Bps* Mip; strain K96243) was produced and purified as described previously (Norville et al., 2011a). The peptidyl-prolyl *cis/trans* isomerase assay was conducted as described previously (Norville et al., 2011b; Begley et al., 2014; Scheuplein et al., 2023) with modifications. Briefly, 20 nM recombinant *Bps* Mip protein was pre-incubated for 6 min at 10°C with SF235, AN_CH_37 or rapamycin at a final concentration of 400 nM or equivalent volume of DMSO in 1.2 ml 35 mM HEPES buffer (pH 7.8) with succinyl-Ala-Phe-Pro-Phe-*p*-nitroanilide (10 mg/ml stock; Bachem #4016001). Chymotrypsin (Merck #C4129) was added to the cuvette at a final concentration of 0.8 mg/ml and mixed. Hydrolysis of the substrate was measured at 390 nm using a Shimadzu 100 UV/visible spectrophotometer at 2 s intervals for 15 min. Three independent runs were performed for each protein and inhibitor/DMSO combination. The inhibition constant (*K_i_*) of SF235 and AN_CH_37 against *Bps* Mip were determined as previously described (Iwasaki et al., 2022; Debowski et al., 2023).

### 2.3 J774A.1 murine macrophage infection assay

J774A.1 murine macrophages were seeded into a 24-well tissue culture treated plate (Thermofisher, #NUN142475) at a concentration of 4 × 10^5^ cells/ml in Dulbecco’s modified Eagle’s medium (DMEM) (Gibco) supplemented with a final concentration of 1% (v/v) GlutaMAX (Gibco, Life Technologies) and 10% (v/v) heat-inactivated fetal calf serum (Gibco, Life Technologies) and incubated for 20 h at 37°C with 5% CO_2_ to achieve a confluent monolayer of approximately 1 × 10^6^ cells/well. *B. pseudomallei* strain K96243 (Dstl, UK) was grown overnight at 37°C for 18 h and adjusted in Leibovitz L-15 medium (Gibco, Life Technologies) supplemented with 1% (v/v) GlutaMAX and 10% (v/v) heat-inactivated fetal calf serum to an absorbance at 580 nm between 0.35 and 0.4 using a PLP Colorimeter. This culture was serially diluted in L-15 medium to achieve a multiplicity of infection (MOI) of approximately 10:1. Prior to infection of cells, *B. pseudomallei* was treated with 50 µM of each inhibitor or DMSO alone for 1 h at 37°C. J774A.1 cells were infected with treated *B. pseudomallei* for 30 min at 37°C. To determine the exact starting inoculum at the time of infection, bacteria were further serially diluted and plated on LB agar. Post-infection, bacteria were aspirated off the cell monolayer, and infected cells were gently washed three times with phosphate-buffered saline (PBS; autoclaved and filter sterilized; Life Technologies) and then incubated with L-15 medium containing 1 mg/ml kanamycin for a further 30 min at 37°C to kill extracellular bacteria. The supernatant was removed, and infected cells were then incubated with L-15 medium containing 250 μg/ml kanamycin with 50 µM of appropriate inhibitor or DMSO for 4 or 24 h at 37°C.

### 2.4 RNA sequencing and data analysis

Cells were stored in RNAProtect Cell Reagent (QIAGEN, #76526) and stored at 4°C overnight or -80°C for long-term storage. Cells from duplicate wells were combined, pelleted and RNA was extracted using the RNeasy Micro Plus kit (QIAGEN, #74034) as per the manufacturer’s protocol. RNA quantity and quality was determined using a NanoDrop 2000 spectrophotometer (Thermo Fisher Scientific). RNA integrity was assessed by Azenta using an RNA ScreenTape (Agilent). Details of each of sample including QC data are provided in **Supplementary Table 1**. Four independent experiments were conducted to obtain four biological replicates for each treatment. Library preparation and RNA sequencing was conducted by Azenta. Briefly, libraries were sequenced using a 1× 150-bp single end rapid run on an Illumina HiSeq platform. To remove technical sequences, including adapters, polymerase chain reaction (PCR) primers, or fragments thereof, and quality of bases lower than 20, pass filter data of fastq format were processed using Trimmomatic (v0.30) (Bolger et al., 2014). Remaining sequence reads were mapped to the *Mus musculus* mm10 reference genome using Hisat2 (v2.0.1) (Kim et al., 2019). Raw data obtained from RNA sequencing were deposited as FASTQ and gene abundance data in Gene Expression Omnibus (GEO) database (accession number: GSE244127).

Gene expression analysis was conducted by Azenta. Briefly, gene expression calculation was performed using published methods (Mortazavi et al., 2008), and the fragments per kilo bases per million reads (FPKM) were calculated. Gene expression results across all samples are available on the GSE244127_all.fpkm_anno.txt.gz file on the GEO database. Expression levels of all genes under different experimental conditions were compared by FPKM profiles. Gene differential analysis was performed using DESeq2 (v1.6.3) and EdgeR (v3.4.6) (Anders and Huber, 2010; Love et al., 2014). The results from EdgeR analysis were further analyzed to determine genes with significant differential expression according to the criteria of absolute fold change greater than 1.5 or less than -1.5 and adjusted *p* value (FDR) less than 0.05. For KEGG pathway analysis, in-house scripts (Azenta) were used to enrich significant differential expression genes. Principal component analysis (PCA) plots were generated using the *R* stats package (v4.3.1). To account for potential batch effects, batch correction was performed using linear models for microarray analysis (limma; v3.56.2) (Ritchie et al., 2015).

### 2.5 Enumeration of viable *B. pseudomallei* in macrophages

At 24 h post-infection with *B. pseudomallei*, macrophage cell monolayers were lysed with MilliQ water (1 mL per well of a 24-well tissue culture plate). The lysed macrophage cells were serially diluted to 10^-4^ (total volume of 1 mL) in PBS before being plated onto LB agar for bacterial enumeration. Colonies were counted after two days incubation at 37°C. Results are presented as the means of seven independent experiments. All statistical analyses were carried out using the Wilcoxon test in Graph-Pad Prism version v8.3.1.

### 2.6 *B. pseudomallei* cytotoxicity assays

*B. pseudomallei*-induced cytotoxicity was assessed through measurement of lactate dehydrogenase (LDH) in cell culture supernatants 24 h post-infection. LDH release was measured using the Roche Cytotoxicity Detection Kit (Roche, Sigma Aldrich #11644793001) as per the manufacturer’s instructions. All results are presented as the means of eight independent experiments. Infected cells treated with DMSO were included as controls. To express the results as the percentage of wildtype cytotoxicity, the LDH activity in SF235 and AN_CH_37 treated wells were compared to that in wells infected with *B. pseudomallei* treated with DMSO. Spontaneous release (LDH release following incubation of uninfected cells with the corresponding inhibitor or DMSO) was subtracted from the final values. All statistical analyses were carried out using the Mann-Whitney *U* test in Graph-Pad Prism version v8.3.1.

### 2.7 Growth of *B. pseudomallei* in broth

Broth microdilutions were tested against three concentrations of SF235 and AN_CH_37 as described previously (Bzdyl et al., 2019; Scheuplein et al., 2023) with modifications. *B. pseudomallei* K96243 was incubated overnight in Luria-Bertani (LB) broth at 37°C with agitation. Overnight cultures were diluted 1:100 in fresh LB broth and were incubated at 37°C for 2 h with agitation. Compounds were diluted across a 96-well plate in LB broth with a final volume of 100 µL at 100 µM. Vehicle control wells were also included with an equivalent volume of DMSO for each concentration (1% DMSO). Following bacterial incubation, 100 µL of *B. pseudomallei* was added to the compound/DMSO containing medium to give a final compound concentration of 50 µM and incubated statically at 37°C for 24 h. *B. pseudomallei* only wells were also included. The optical density of plates was read at 590 nm using a spectrophotometer (Bio-Rad Xmark). Background absorbance of media with inhibitor was subtracted from all wells. Results are expressed as the percentage of growth relative to the unexposed *B. pseudomallei* control wells using the equation below:


$$\text{\% growth}=\;\frac{\text{inhibitor exposed }B.\;pseudomallei\text{ OD}590}{\text{unexposed }B.\;pseudomallei\text{ OD}590}\text{ x }100$$

Greater than 20% growth was considered viable. Results are presented as the means of three independent experiments. All statistical analyses were carried out using the Wilcoxon test in Graph-Pad Prism version v8.3.1.

### 2.8 Enzyme-linked immunosorbent substrate assays of secreted cytokines

IL-1β, IL-6, TNFα and MCP-1/CCL2 were detected using standardized sandwich ELISAs in cell culture supernatants from J774A.1 cells 24 hours following infection with *B. pseudomallei* following the protocol described above. Cytokine measurements on supernatants from uninfected J774A.1 cells treated with DMSO were also performed. Cytokines were quantitated using Invitrogen uncoated ELISAs (Thermo Fisher Scientific #88-7013-86, 88-7391-88, 88-7324-86, 88-7064-86) as per the manufacturer’s instructions. Results were determined by measuring absorbance at 450 nm and 570 nm using a Bio-Rad Xmark plate reader. The values of 570 nm were subtracted from those of 450 nm and unknown values were interpolated into standard curves in Prism v8.3.1 using the second order polynomial equation. Results are presented as the means of eight independent experiments. All statistical analyses were carried out using Wilcoxon test in Graph-Pad Prism version v8.3.1.

## 3 Results

### 3.1 The PPIase activity of *B. pseudomallei* Mip is inhibited by small molecule compounds SF235 and AN_CH_37

**Figure 1:**
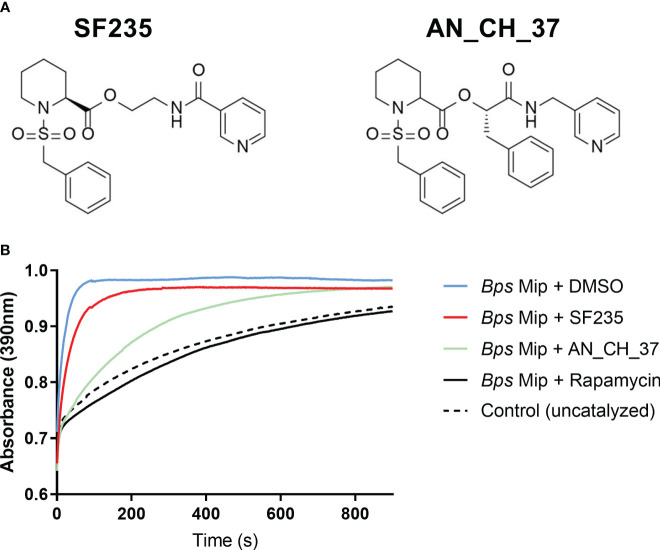
Inhibition of PPIase activity of recombinant Mip with small molecule compounds, SF235 and AN_CH_37. **(A)** Structures of pipecolic acid-based Mip inhibitors, SF235 and AN_CH_37. The synthesis of these inhibitors have been described elsewhere (Seufert et al., 2016; Scheuplein et al., 2023). **(B)** A protease-coupled PPIase assay was used to measure *cis/trans* isomerization of the substrate succinyl-Ala-Phe-Pro-Phe-*p*-nitroanilide in the presence of 20 nM recombinant *B. pseudomallei* Mip protein and vehicle control (DMSO). Inhibition of PPIase activity was measured with the addition of 400 nM of SF235, AN_CH_37 and rapamycin. An uncatalyzed control reaction with no Mip protein was also included. *n* = 3.

SF235 and AN_CH_37 are pipecolic acid derivatives that contain the pipecolic sulphonamide and an amide-based linker to a pyridyl unit. In contrast to SF235, AN_CH_37 contains an additional benzyl moiety which has been shown to increase stability and potency [**Figure 1A**; (Scheuplein et al., 2023)]. To verify that SF235 and AN_CH_37 target and directly inhibit the Mip protein in *B. pseudomallei* (*Bps* Mip), the inhibition of the PPIase activity of recombinant *Bps* Mip was initially evaluated in a protease-coupled assay by measuring *cis*-*trans* isomerization of the tetrapeptide Suc-Ala-Phe-Pro-Phe-*p*-nitroanilide. These studies demonstrated that at a concentration of 400 nM, AN_CH_37 inhibited the activity of *Bps* Mip at a greater rate than SF235 (**Figure 1B**). The PPIase activity of *Bps* Mip was completely abolished by the known PPIase inhibitor rapamycin. Further analysis revealed the *K_i_* values of SF235 and AN_CH_37 to be 290 ± 60 (Iwasaki et al., 2022) and 180 ± 30 nM, respectively. We have previously determined the *Ki* of rapamycin against *Bps* Mip to be 3 ± 2 nM (Norville et al., 2011a).

### 3.2 Global transcriptional profiling of murine macrophage J774A.1 cells infected with *B. pseudomallei* in the presence and absence of SF235 and AN_CH_37

The murine macrophage J774A.1 cell line has been utilized to study the innate immune response to bacterial pathogens (Cross et al., 2004; Yu et al, 2010). Indeed, in *in vitro* models of melioidosis, we and others have demonstrated that *B. pseudomallei* infects, survives and replicates intracellularly in these cells (Miyagi et al, 1997; Harley et al., 1998; Sim et al., 2009; Rasko and Sperandio, 2010; Norville et al., 2011b; Srinon et al., 2013; Ritchie et al., 2015; Reimer et al., 2016; Seufert et al., 2016; Pandey et al., 2017; Bzdyl et al., 2019; Sangsri et al., 2020; Scheuplein et al., 2023). Despite this, the inflammatory response following *B. pseudomallei* infection has not been studied. In this respect, J774A.1 cells were used to characterize the global host gene expression response to *B. pseudomallei* and to determine whether inhibition of the virulence factor Mip results in modifications of the inflammatory responses to infection. To do this, we firstly performed transcriptional analysis on cells infected with *B. pseudomallei* K96243 in the presence of the vehicle control DMSO and compared to that of uninfected cells treated with DMSO. We then evaluated the global transcript profiles of cells infected with *B. pseudomallei* in the presence of SF235 or AN_CH_37 and compared it to that of *B. pseudomallei* infected cells treated with DMSO. These analyses were performed four hours following infection by RNA-sequencing to characterize the early stages of infection. The RNA quality and data alignment statistics are shown in **Supplementary Table 1**. RNA extracted from cells 8, 12, and 24 hours post-infection resulted in insufficient quantity and/or quality of RNA required for RNA-seq in *B. pseudomallei* infected samples and thus not used for downstream analyses (data not shown).

**Figure 2:**
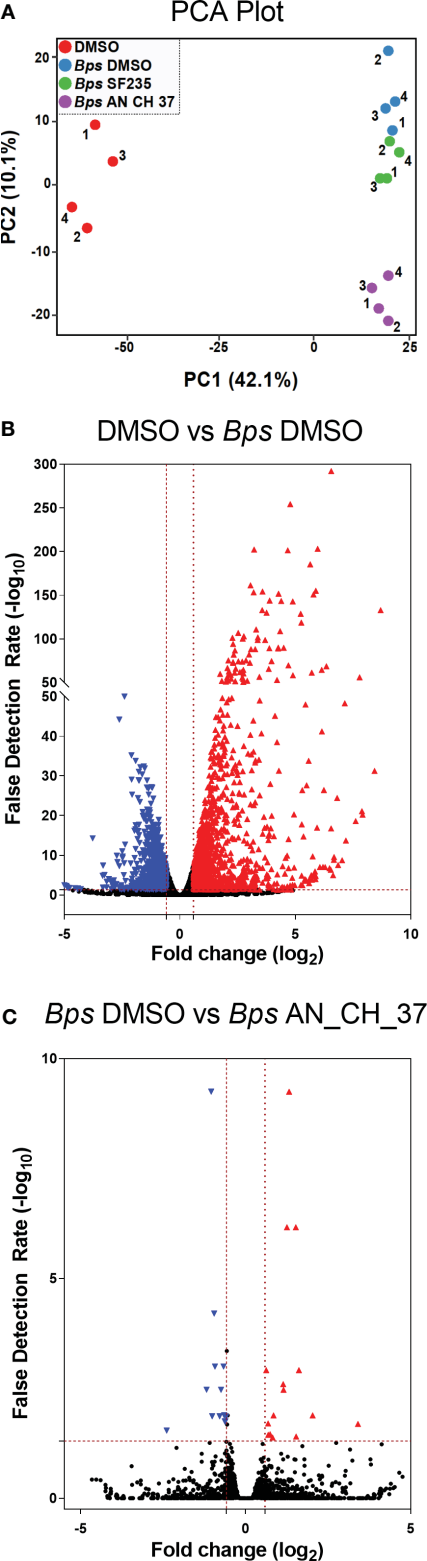
Principle component analysis (PCA) and Differential Gene Expression analysis of murine macrophages following *B. pseudomallei* infection in the presence and absence of Mip inhibitors, and uninfected control using RNA-seq four hours post-infection. **(A)** PCA plot. Each dot (1-4) represents one of four biological replicates for each treatment. **(B)** Differential expression volcano plots of *B pseudomallei* infected cells compared to uninfected cells with vehicle (DMSO) control. **(C)** Differential expression volcano plots of *B pseudomallei* infected cells in the presence of Mip inhibitor AN_CH_37 compared to infected DMSO control. Red triangles represent genes that are significantly upregulated and blue triangles represent those that are significantly downregulated. The number of differentially expressed genes with the criteria of absolute fold change (FC) greater than 1.5 (red) and less than -1.5 (blue) and absolute false detection rate (FDR)< 0.05 was determined as significant, as represented by the red dotted lines. Volcano plots represents fold change (log_2_) of gene expression and the FDR of the differential expression in log_10_ (-log_10_ FDR).

Visualization of overall similarities between the global transcriptional expression patterns four hours post-infection by Principal Component Analysis (PCA) showed complete separation between uninfected macrophages treated with DMSO and *B. pseudomallei* infected cells on the first principal component (**Figure 2A**). After removing batch effects (**Supplementary Figure 1**), a distinction in infected cells treated with AN_CH_37 compared to DMSO and SF235 was evident on the second principal component (**Figure 2A**). 1229 upregulated and 1086 downregulated differentially expressed genes (DEGs) were identified in *B. pseudomallei* infected cells with DMSO compared to uninfected cells according to the criteria of absolute fold change greater or less than 1.5 and -1.5, respectively, and a false discovery rate (FDR) of less than 0.05 (**Figure 2B**; **Table 1**; **Supplementary Table 2**). When comparing the effect of the Mip inhibitors on the response to *B. pseudomallei* infection under the same statistical analysis conditions, we observed no DEGs with SF235 treatment (**Supplementary Table 3**), while treatment with AN_CH_37 resulted in 16 upregulated and 12 downregulated differentially expressed genes (**Figure 2C**; **Table 2**; **Supplementary Table 5**).

**Table 1:** List of top 30 differentially expressed genes following infection with *B. pseudomallei* in J774A.1 macrophages ranked by fold change (with a false detection rate (FDR) less than 0.05).

Gene Symbol	Fold change	FDR	id
*Il1a*	2,302	1.78E-30	ENSMUSG00000027399
*Il1b*	1,170.3	3.55E-53	ENSMUSG00000027398
*Cxcl2*	412.4	1.36E-133	ENSMUSG00000058427
*Il12b*	343.2	5.38E-32	ENSMUSG00000004296
*Csf2*	239.9	6.26E-21	ENSMUSG00000018916
*Cxcl1*	235.5	7.64E-22	ENSMUSG00000029380
*Nos2*	219.1	1.17E-56	ENSMUSG00000020826
*Ptgs2*	208.5	0	ENSMUSG00000032487
*Htra1*	195	2.47E-19	ENSMUSG00000006205
*Xirp2*	145.6	1.93E-14	ENSMUSG00000027022
*Lif*	140.7	5.07E-49	ENSMUSG00000034394
*Ccl20*	130.2	1.92E-09	ENSMUSG00000026166
*Heatr9*	115.1	2.74E-08	ENSMUSG00000018925
*Il6*	111.7	3.53E-25	ENSMUSG00000025746
*Ptprg*	102	2.86E-10	ENSMUSG00000021745
*Gm25360*	101.4	1.41E-09	ENSMUSG00000094655
*Snord118*	99.1	1.09E-05	ENSMUSG00000064899
*Tnf*	93.7	6.05E-293	ENSMUSG00000024401
*Nr4a3*	93.3	1.66E-17	ENSMUSG00000028341
*Pkp1*	92.4	4.49E-07	ENSMUSG00000026413
*Tmem88*	89.2	1.80E-07	ENSMUSG00000045377
*Acod1*	81.2	2.27E-69	ENSMUSG00000022126
*Sprr2e*	77.9	9.79E-08	ENSMUSG00000055030
*Saa3*	75.4	3.70E-27	ENSMUSG00000040026
*Fam71a*	73	8.80E-08	ENSMUSG00000091017
*Il23a*	70.8	6.50E-42	ENSMUSG00000025383
*Ccl7*	70.4	2.62E-65	ENSMUSG00000035373
*Fam166a*	66.5	9.49E-08	ENSMUSG00000026969
*Nfkbiz*	62.4	6.59E-204	ENSMUSG00000035356
*Edn1*	61.2	1.13E-17	ENSMUSG00000021367

Gene Symbol: Gene name or description; Fold change: the absolute fold change converted from Log_2_ fold change; FDR: the adjusted *p* values; id: gene ID. Analysis was performed by comparing *B. pseudomallei* infected macrophages treated with DMSO to macrophages treated with DMSO only.

**Table 2:** List of differentially expressed genes following infection with *B. pseudomallei* in J774A.1 macrophages in the presence of Mip inhibitor AN_CH_37 ranked by fold change (with false detection rate (FDR) less than 0.05).

Gene Symbol	Fold change	FDR	id
*Il1b*	-5.3	0.029	ENSMUSG00000027398
*Zfyve9*	-2.3	0.003	ENSMUSG00000034557
*Abca1*	-2.1	5.64E-10	ENSMUSG00000015243
*Mfap1b*	-2	0.014	ENSMUSG00000048222
*Trem1*	-1.9	6.31E-05	ENSMUSG00000042265
*Ptgs2*	-1.9	0.001	ENSMUSG00000032487
*Fosl2*	-1.7	0.014	ENSMUSG00000029135
*Tnrc6a*	-1.7	0.003	ENSMUSG00000052707
*Rbm25*	-1.6	0.001	ENSMUSG00000010608
*Trib1*	-1.6	0.013	ENSMUSG00000032501
*Brwd3*	-1.6	0.018	ENSMUSG00000063663
*Pde4b*	-1.5	0.018	ENSMUSG00000028525
*Myo9a*	-1.5	0.014	ENSMUSG00000039585
*Rbbp6*	-1.5	0.001	ENSMUSG00000030779
*Setx*	-1.5	0.021	ENSMUSG00000043535
*Osm*	-1.5	0.013	ENSMUSG00000058755
*H1f0*	1.5	0.001	ENSMUSG00000096210
*Arrdc3*	1.6	0.02	ENSMUSG00000074794
*Crebzf*	1.6	0.036	ENSMUSG00000051451
*Snhg12*	1.7	0.035	ENSMUSG00000086290
*Trib3*	1.8	0.042	ENSMUSG00000032715
*1810026B05Rik*	1.8	0.013	ENSMUSG00000101970
*Grhl2*	2.2	0.003	ENSMUSG00000022286
*5430416N02Rik*	2.2	0.003	ENSMUSG00000097772
*Hist1h2be*	2.4	6.86E-07	ENSMUSG00000047246
*Hist1h1d*	2.5	5.64E-10	ENSMUSG00000052565
*Hist1h1b*	2.9	6.86E-07	ENSMUSG00000058773
*Gpa33*	2.9	0.04	ENSMUSG00000000544
*Gm42743*	3.1	0.001	ENSMUSG00000050936
*Gm19590*	4.1	0.013	ENSMUSG00000107061
*Usp17le*	10.6	0.020	ENSMUSG00000043073

Gene Symbol: Gene name or description; Fold change: the absolute fold change converted from Log_2_ fold change; FDR: the adjusted *p* values; id: gene ID. Analysis was performed by comparing *B. pseudomallei* infected macrophages treated with AN_CH_37 to *B. pseudomallei* infected macrophages treated with DMSO.

**Figure 3:**
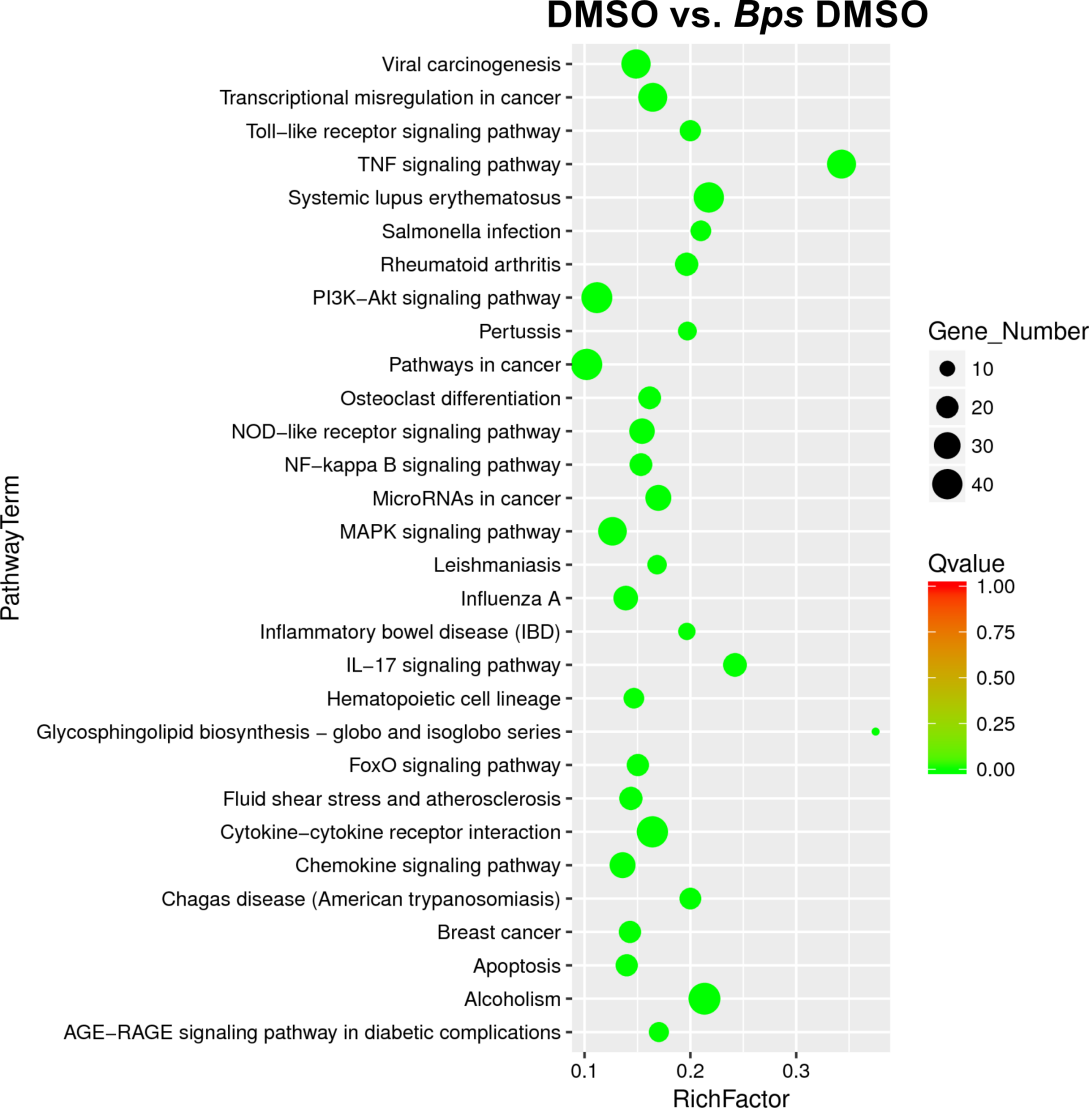
Scatter plot of differential gene KEGG enrichment following infection with *B. pseudomallei* in the presence of DMSO (vehicle control of Mip inhibitors). The degree of KEGG enrichment is measured by the Richfactor (ratio of the number of genes differentially expressed in the pathway to the total number of genes in the pathway), Qvalue (adjusted *p* value after multiple hypothesis testing), and the Gene Number (the number of differential genes in the pathway). The top 30 pathways that are most significantly enriched for the analysis are presented.

Examination of the top significantly expressed genes following *B. pseudomallei* infection revealed upregulation of a vast array of immune associated genes, with *il1a*, *il1b* and *cxcl2* constituting the most strikingly upregulated DEGs in infected macrophages compared to uninfected cells based on fold change (**Table 1**). At this time point, *tlr2* was also highly upregulated (fold change = 7.3; FDR = 3.58 x 10^-76^). The complete list of differentially expressed genes is provided in **Supplementary Table 2**. To facilitate biological interpretation of the large number of genes altered in expression upon infection, pathway functional enrichment analysis was conducted using KEGG (Kyoto Encyclopedia of Genes and Genomes). The KEGG enrichment analysis is represented in **Figure 3** in which the top 30 pathways that were most significantly enriched are presented. Enrichment was assessed using “RichFactor” which was defined as the ratio of the number of genes differentially expressed in the pathway to the total number of genes in the pathway. Thus, the larger the RichFactor, the greater the degree of enrichment observed. Specifically, this analysis showed significant enrichment of the ‘TNF signaling pathway’ (adjusted *p* value = 1.41 x 10^-18^) with 36 (7.35%) DEGs within this pathway (**Supplementary Table 4**). ‘Cytokine-cytokine receptor interaction’, ‘IL-17 signaling’, ‘NOD-like receptor signaling’ and ‘Toll-like receptor signaling’ (adjusted *p* values of 9.90 x 10^-10^, 8.29 x 10^-9^, 5.95 x 10^-6^ and 6.02 x 10^-6^, respectively) constituted the next most significant immune-related pathways identified.

To determine the effect of treatment with Mip inhibitors on differential gene expression of J774A.1 cells infected with *B. pseudomallei*, we identified DEGs comparing SF235 or AN_CH_37 infected cells with DMSO treatment only. Although SF235 treatment did not reveal any DEG with an FDR less than 0.05 (**Supplementary Table 3**), treatment with AN_CH_37 resulted in significant downregulation of *il1b* (fold change = -5.3; FDR = 0.03) and upregulation of a deubiquitinating enzyme *usp17le* (fold change = 10.6; FDR = 0.02). The list of the top differentially expressed genes is shown in **Table 2** with the complete list provided in **Supplementary Table 5**.

### 3.3 AN_CH_37 treatment dampens the production of pro-inflammatory cytokines, cytotoxicity and intracellular bacterial burden in *B. pseudomallei* infected macrophages

**Figure 4:**
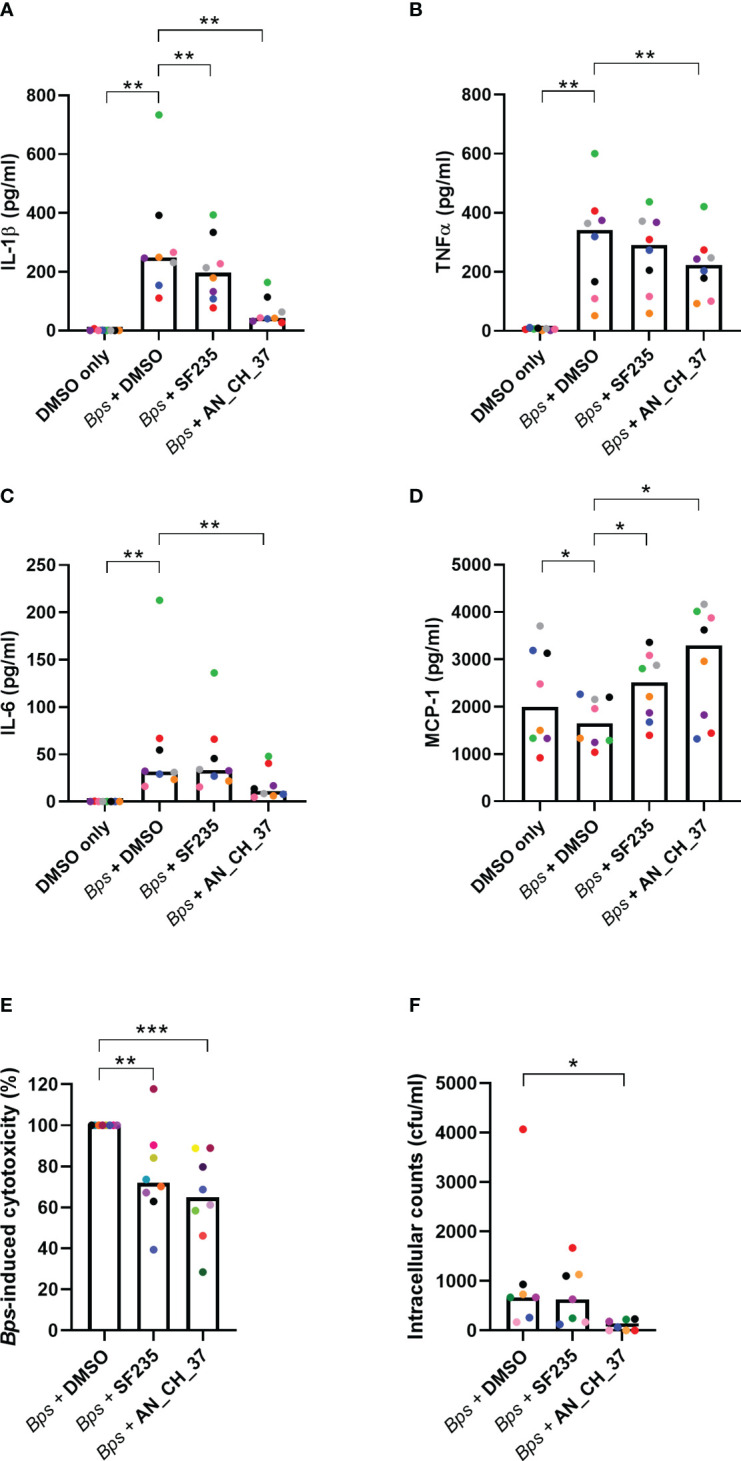
Cytokine secretion profiles, cytotoxicity and intracellular bacterial burden in J7741.A macrophages infected with *B. pseudomallei* in the presence and absence of Mip inhibitors, SF235 and AN_CH_37, 24 hours post-infection. Secretion of **(A)** IL-1β, **(B)** TNFα, **(C)** IL-6 and **(D)** MCP-1 were measured in supernatants harvested after 24 h of infection by ELISA. **(E)** *B. pseudomallei* induced cytotoxicity as determined by LDH release in supernatants. **(F)** Intracellular bacterial survival (colony forming units; cfu). Mean and each biological replicate are shown. Significant differences in relation to the uninfected vehicle control (DMSO) or *B. pseudomallei* treated with DMSO are denoted (∗*p* ≤ 0.05; ∗∗*p* ≤ 0.01, ∗∗∗*p* ≤ 0.001, Wilcoxon test for **(A–D, F)** and Mann-Whitney *U* test for **(E)**).

According to the transcriptional evaluation, *B. pseudomallei* induces the expression of innate immune genes and key inflammatory pathways early in infection, and AN_CH_37 treatment downregulates some of these genes, particularly *il1b*. To validate this, the production of the pro-inflammatory cytokines IL-1β, TNF-α and IL-6 were measured in cell culture supernatants using ELISA 24 hours post-infection (**Figures 4A–C**). *B. pseudomallei* induced IL-1β, TNF-α and IL-6 production as anticipated, which in the presence of AN_CH_37 was significantly decreased (*p*< 0.01). On the other hand, treatment with SF235 resulted in a reduction in IL-1β only (*p*< 0.01). To investigate the potentially protective role of the chemokine, monocyte chemotactic protein-1 (MCP-1), we measured MCP-1 in the cell culture supernatants and found that *B. pseudomallei* infection resulted in a significant reduction in MCP-1, whereas MCP-1 production was higher with SF235 or AN_CH_37 treatment (*p*< 0.05; **Figure 4D**).

To examine cell death and *B. pseudomallei*-induced cell cytotoxicity, LDH was measured in cell culture supernatants in the presence and absence of Mip inhibitors. As reported previously, SF235 (Iwasaki et al., 2022) and for the first time, AN_CH_37 significantly reduced LDH release following *B. pseudomallei* infection (*p*< 0.001; **Figure 4E**). The cytotoxicity of Mip inhibitors alone, to macrophage cells, were low (mean 14.42% for SF235 and 12.54% for AN_CH_37; **Supplementary Figure 2**). When bacteria were enumerated at the same time point, a significant difference in the number of intracellular bacteria was seen with AN_CH_37 (*p*< 0.05) but not SF235, demonstrating reduced survival and/or replication in macrophage cells following treatment with AN_CH_37 (**Figure 4F**). Neither SF235 nor AN_CH_37 treatment reduced planktonic bacterial growth (**Supplementary Figure 3**).

## 4 Discussion

Targeting virulence factors represents a novel therapeutic strategy to combat bacterial infections (Rasko and Sperandio, 2010; Allen et al., 2014), particularly against multidrug resistant bacteria such as *Burkholderia pseudomallei*. Here, we demonstrate that a small molecule compound exerts no inhibitory effects on planktonic bacterial growth but by inhibiting the virulence factor Mip in *B. pseudomallei*, they indirectly assist macrophages to kill the pathogen that would otherwise be present at a higher intracellular burden. Treatment with the Mip inhibitor also resulted in the attenuation of cell-based inflammatory responses, namely pro-inflammatory cytokines IL-1β, TNFα and IL-6 (Storek and Monack, 2015), as well as *B. pseudomallei* induced cytotoxicity in murine macrophages *in vitro*. Therefore, the results presented in this study outline the therapeutic promise of the Mip inhibitor, AN_CH_37 in treating melioidosis.

The potential role of antivirulence strategies as an alternative to traditional antibiotics to reduce antimicrobial resistance has been discussed in detail in recent years (Clatworthy et al, 2007; Rasko and Sperandio, 2010; Allen et al., 2014) with the concept being to disarm the pathogen and enable the host immune system to clear the bacteria. We focused on the macrophage infectivity potentiator (Mip) protein, a protein folding enzyme that exhibits peptidyl-prolyl isomerases (PPIase) activity, due to its established role in *B. pseudomallei* pathogenesis (Norville et al, 2011b). Additionally, Mip has been shown to be a virulence factor in other bacterial species including, *Legionella pneumophila*, *Neisseria gonorrhoeae*, *Neisseria meningitidis* and *Coxiella burnetii*, as well as parasites *Trypanosoma cruzi* and *Leishmania major* (Cianciotto and Fields, 1992; Lundemose et al, 1993; Moro et al., 1995; Leuzzi et al., 2005; Reimer et al., 2016; Iwasaki et al., 2022; Debowski et al., 2023) Since it has been shown that other PPIase proteins have immunomodulatory roles during bacterial infections (Pandey et al., 2017), we investigated, for the first time, the possible involvement of Mip in the innate immune response to *B. pseudomallei* infection. Using small molecule inhibitors designed to inhibit Mip, we demonstrated that treatment of *B. pseudomallei*-infected murine macrophages with the inhibitor AN_CH_37 resulted in a decrease in secreted pro-inflammatory cytokines IL-1β, TNFα and IL-6, and an increase in MCP-1, 24 hours post-infection. A significant downregulation of *il1b* was evident four hours post-infection as determined by RNA sequencing. Treatment with SF235, which does not contain an additional benzyl moiety that AN_CH_37 possesses did not result in any significant changes to the host transcriptome. It is worth noting that AN_CH_37 was developed as an improvement to SF235, whereby the benzyl group in the linker chain was introduced onto SF235 to generate AN_CH_37 to increase chemical stability of the pipecolic ester, as well as to increase the inhibitory potency by occupying an additional binding pocket to enhance the affinity to the *B. pseudomallei* Mip protein (Scheuplein et al., 2023). The efficiency with which AN_CH_37 treatment suppressed the pro-inflammatory response suggests that Mip supports potent stimulation of IL-1β, TNFα and IL-6, and thus may play an important role in host inflammatory pathology following infection.

Pro-inflammatory immune responses are a critical component of the host response to pathogens. However, excessive inflammation can also cause damage to the host. It has been postulated that the development of acute melioidosis is due to an excess of inflammation (Tan et al., 2010). Indeed, analyses of serum cytokine expression in patients with serious melioidosis have shown increased concentrations of pro-inflammatory cytokines such as IFN-γ (Lauw et al., 1999) and IL-6 (Lauw et al., 1999). Serum IL-6, along with the anti-inflammatory cytokine IL-10, has also been identified as a predictor of mortality (Lauw et al., 1999). Since macrophages and monocytes are the main source of cytokines such as TNF, IL-1, IL-6, IL-8, and IL-12 and the production of these cytokines in concert with other mediators can produce systemic effects such as fever (Duque and Descoteaux, 2014), suppressing macrophage proinflammatory production is likely to have overall clinical benefits. Our data support this idea *in vitro*, whereby treatment with AN_CH_37 also resulted in a reduction in cell death as measured by lactate dehydrogenase and intracellular bacterial burden. The findings from this study thus warrants further efficacy studies of Mip inhibitors and provides the basis for lead optimization towards *in vivo* delivery.

Inhibition of Mip had the most profound effect on IL-1β production. Previous work by Ceballos-Olvera et al. revealed that the NLRP3 inflammasome and its downstream product IL-1β is detrimental to the host due to excessive recruitment of neutrophils, which may support intracellular bacterial growth, tissue damage, and inhibition of IFN-γ production in a murine model of melioidosis (Ceballos-Olvera et al., 2011). More recently, anti-IL-1β treatment has been shown to protect mice against *B. pseudomallei* infection (Weehuizen et al., 2016). In concordance with these studies, we speculate that IL-1β secretion is deleterious to murine macrophages, however the mechanisms underlying Mip inhibition and reduction in IL-1β production, cell death and bacterial burden remains to be determined. RNA-seq analysis showed upregulation of *nlrp3* and *il1b* gene expression four hours post-infection suggesting that the NLRP3 inflammasome mediates the release of IL-1β. The potentially protective role of IL-18 was not studied as IL-18 was not measured in cell culture supernatants and *il18* was not differentially regulated in response to *B. pseudomallei* infection. Instead, our results highlighted a protective role of mononuclear cell attractant, MCP-1, whereby Mip inhibition resulted in an increase in MCP-1 secretion. Induction of MCP-1 has been documented in murine pulmonary epithelial cells (Sim et al., 2009) and in the lungs and spleens of mice challenged intranasally with *B. pseudomallei* (Easton et al., 2007; Conejero et al., 2011). However, a protective role of MCP-1 has only been demonstrated in *Burkholderia mallei* (Goodyear et al., 2010).

Treatment with AN_CH_37 also resulted in an upregulation of *usp17la* with a 10-fold change from untreated infected macrophages. USP17la, ubiquitin carboxyl-terminal hydrolase 17-like protein A, is a deubiquitinating enzyme that is part of the USP17 (or DUB3) subfamily of ubiquitin-specific proteases (USPs). There is increasing evidence to support that USPs plays crucial roles in the control of proinflammatory immune pathways (Lopez-Castejon and Edelmann, 2016). Specifically, USP17 has been implicated in the regulation of cell proliferation and survival (Burrows et al., 2004). It has also been shown that USP17 is induced in response to IL-4 and IL-6 stimulation, which suggests a role in regulating immune function (Burrows et al., 2004). Further studies are required to validate and elucidate a potentially protective role of USP17la in *B. pseudomallei* infection. However, given the importance of other deubiquitinases in the NF-κB pathway and inflammasome activation (Lopez-Castejon and Edelmann, 2016), it may be activated to regulate the inflammatory response in macrophages.

RNA-seq analysis was utilized to demonstrate gene regulation in murine macrophages four hours following infection with *B. pseudomallei*. Of particular interest, we were able to gain an insight into the potential roles of non-secretory proteins in the inflammatory response. For example, we found a significant upregulation of *tlr2* in *B. pseudomallei* infected J774A.1 cells in comparison to uninfected cells, highlighting the participation of TLR2 in the early immune response against infection. The high expression level of *tlr2* is similar to that reported by Feterl et al. in RAW264.7 macrophages two hours post-infection (Feterl et al, 2008). Other studies have reported a critical role of TLR2 *in vitro* and *in vivo* (Wiersinga et al., 2007; West et al., 2008). In addition to the activation of TLR signaling pathways, differential gene KEGG enrichment analysis also revealed a profound upregulation of genes in the TNF signaling pathway following infection. We validated this in the context of TNF-α release from macrophages and indeed demonstrated increase in TNF-α in cell culture supernatants following infection, which was significantly reduced with AN_CH_37. The dual role of TNF-α in *B. pseudomallei* infection has been implicated in both defense and susceptibility control (Mariappan et al., 2021), however since increased TNF-α levels in human melioidosis sepsis patients have been linked to higher mortality rates (Cheng and Currie, 2005), there are likely benefits to reducing levels.

In conclusion, we provide evidence that an antivirulence approach targeting *B. pseudomallei* Mip attenuates pro-inflammatory responses, *B. pseudomallei*-induced cytotoxicity and bacterial burden in murine macrophages *in vitro*. These results clearly highlight a novel therapeutic approach to reduce potentially damaging pro-inflammatory host responses. This may serve as an alternative to, or be utilized in combination with, traditional antibiotics to reduce the burden of infectious diseases caused by intracellular pathogens such as *B. pseudomallei*.

## Data availability statement

The datasets generated for this study can be found in the GEO repository. https://www.ncbi.nlm.nih.gov/geo/query/acc.cgi?acc=GSE244127.

## Ethics statement

Ethical approval was not required for the studies on animals in accordance with the local legislation and institutional requirements because only commercially available established cell lines were used.

## Author contributions

JI: Conceptualization, Data curation, Formal analysis, Funding acquisition, Investigation, Methodology, Project administration, Resources, Supervision, Validation, Visualization, Writing – original draft, Writing – review & editing. NB: Investigation, Writing – review & editing. DL-S: Investigation, Writing – review & editing. NS: Investigation, Writing – review & editing. MD: Data curation, Formal analysis, Resources, Visualization, Writing – review & editing. ED: Data curation, Writing – review & editing. NH: Investigation, Resources, Writing – review & editing. UH: Funding acquisition, Resources, Supervision, Writing – review & editing. MS-T: Conceptualization, Funding acquisition, Project administration, Resources, Supervision, Writing – review & editing

## Supplementary material

Supplementary Figure 1 Quality control measures for the RNA-Seq data set. **(A)** Boxplot of log_2_ transformed samples shows equal loading. The bottom and the top of the boxes correspond to the 25th (Q1) and 75th (Q3) percentiles, and the internal band is the 50th percentile (median). The plot whiskers represent the 95% confidence intervals shown down to the minimum (Q1-1.5*IQR) and up to the maximum (Q3 +1.5*IQR) value. IQR= interquartile range. Outside points correspond to potential outliers. **(B)** Pearson correlation heatmap among the log_2_ transformed samples shows high reproducibility between samples. **(C)** Principal component analysis (PCA) plots before (left) and after removing batch effects (right).

Supplementary Figure 2 Inhibitor-induced cytotoxicity. Cytotoxicity was measured after J774A.1 macrophage cells were incubated with SF235 (50 μM), AN_CH_37 (50 μM) or control (1% DMSO) for 24 hours using Roche LDH Cell Cytotoxicity kit. n=8 for SF235 and AN_CH_37; n=16 for DMSO.

Supplementary Figure 3 Growth kinetics of *B. pseudomallei* in the presence and absence of Mip inhibitor SF235, AN_CH_37 and vehicle control (DMSO) in Luria Broth. Values are the mean MICs from three biological replicates. MIC was determined by measuring the optical density at 590 nm at 24 hours after DMSO or inhibitor exposure, and the MIC was called the lowest concentration that resulted in less than 20% growth of the unexposed control on that plate. n=8 for SF235 and AN_CH_37; n=16 for DMSO.

Supplementary Table 1 RNA quality and data alignment statistics. Sample ID, Grouping, RNA quality, total reads and total mapped are given.

Supplementary Table 2 List of differentially expressed genes when comparing *B. pseudomallei* infected J774A.1 macrophages treated with DMSO with uninfected J774A.1 macrophages treated with DMSO. Only genes that were significantly differentially expressed as defined by false detection rate (FDR) < 0.05 and absolute log fold change (FC) < or > 1.5 (equivalent to log_2_FC < or > 0.585) is presented. The input data for gene differential expression is the read count data obtained from gene expression analysis. (1) Id: gene ID (2) logFC: the log_2_ of the fold change (3) logCPM: the average log_2_-counts-per-million (4) PValue: calculated probability (5) FDR: the adjusted p values (6) GeneSymbol: Gene name or description (7) GO: Gene ontology ID.

Supplementary Table 3 List of differentially expressed genes when comparing *B. pseudomallei* infected J774A.1 macrophages treated with Mip inhibitor SF235 with *B. pseudomallei* infected J774A.1 macrophages treated with DMSO. The input data for gene differential expression is the read count data obtained from gene expression analysis. (1) Id: gene ID (2) logFC: the log_2_ of the fold change (3) logCPM: the average log_2_-counts-per-million (4) PValue: calculated probability (5) FDR: the adjusted *p* values (6) GeneSymbol: Gene name or description (7) GO: Gene ontology ID.

Supplementary Table 4 Differential Gene KEGG Enrichment List when comparing *B. pseudomallei* infected J774A.1 macrophages treated with DMSO with uninfected J774A.1 macrophages treated with DMSO. (1) PathwayID: pathway ID (2) Pathway: path name (3) Category (4) DEGs with pathway annotation: the number of differentially expressed genes in the pathway (5) All genes with pathway annotation: the number of all the genes in the pathway (6) RichFactor: the ratio of the number of genes differentially expressed in the pathway to the total number of genes in the pathway (7) Pvalue: *p* value of the pathway enrichment (8) Qvalue: q value of the pathway enrichment (9) Genelist: list of genes enriched in this pathway (10) Pathwaylink: web link to KEGG Pathway Maps for each pathway.

Supplementary Table 5 List of differentially expressed genes when comparing *B. pseudomallei infected* J774A.1 macrophages treated with Mip inhibitor AN_CH_37 with *B. pseudomallei* infected J774A.1 macrophages treated with DMSO. Only genes that were significantly differentially expressed as defined by false detection rate (FDR) < 0.05 and absolute log fold change (FC) < or > 1.5 (equivalent to log_2_FC < or > 0.585) is presented. The input data for gene differential expression is the read count data obtained from gene expression analysis. (1) Id: gene ID (2) logFC: the log_2_ of the fold change (3) logCPM: the average log_2_-counts-per-million (4) PValue: calculated probability (5) FDR: the adjusted *p* values (6) GeneSymbol: Gene name or description (7) GO: Gene ontology ID.
