# Supplementary figures and images for: Inhibition of macrophage infectivity potentiator in Burkholderia pseudomallei suppresses pro-inflammatory responses in murine macrophages
Source: Front Cell Infect Microbiol. 2024 Mar 25;14:1353682. doi: 10.3389/fcimb.2024.1353682 (PMC10999550; doi:10.3389/fcimb.2024.1353682)

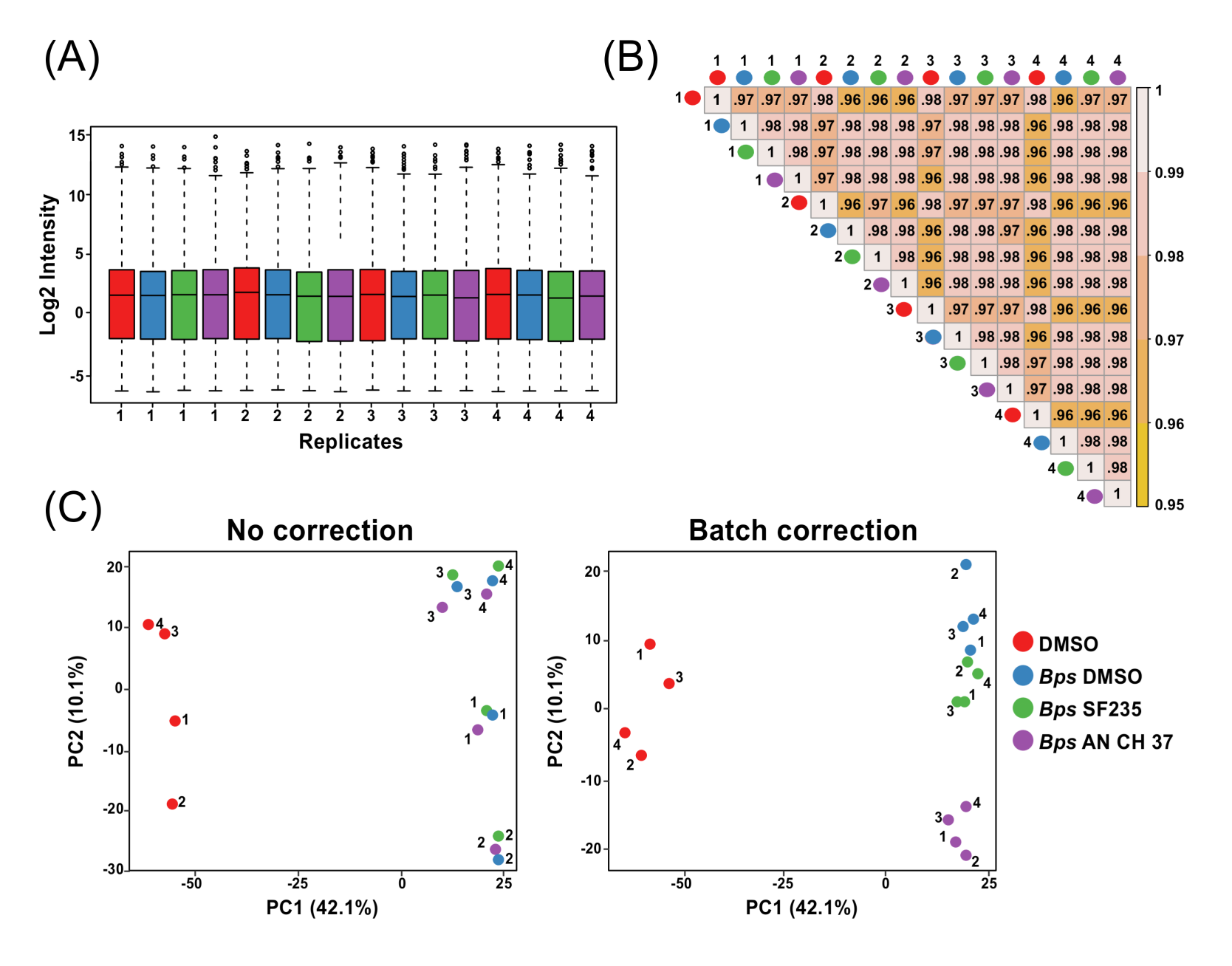

Supplement: Supplementary Figure 1 — Quality control measures for the RNA-Seq data set. (A) Boxplot of log2 transformed samples shows equal loading. The bottom and the top of the boxes correspond to the 25th (Q1) and 75th (Q3) percentiles, and the internal band is the 50th percentile (median). The plot whiskers represent the 95% confidence intervals shown down to the minimum (Q1-1.5*IQR) and up to the maximum (Q3 +1.5*IQR) value. IQR= interquartile range. Outside points correspond to potential outliers. (B) Pearson correlation heatmap among the log2 transformed samples shows high reproducibility between samples. (C) Principal component analysis (PCA) plots before (left) and after removing batch effects (right). [file Image_1.tif]

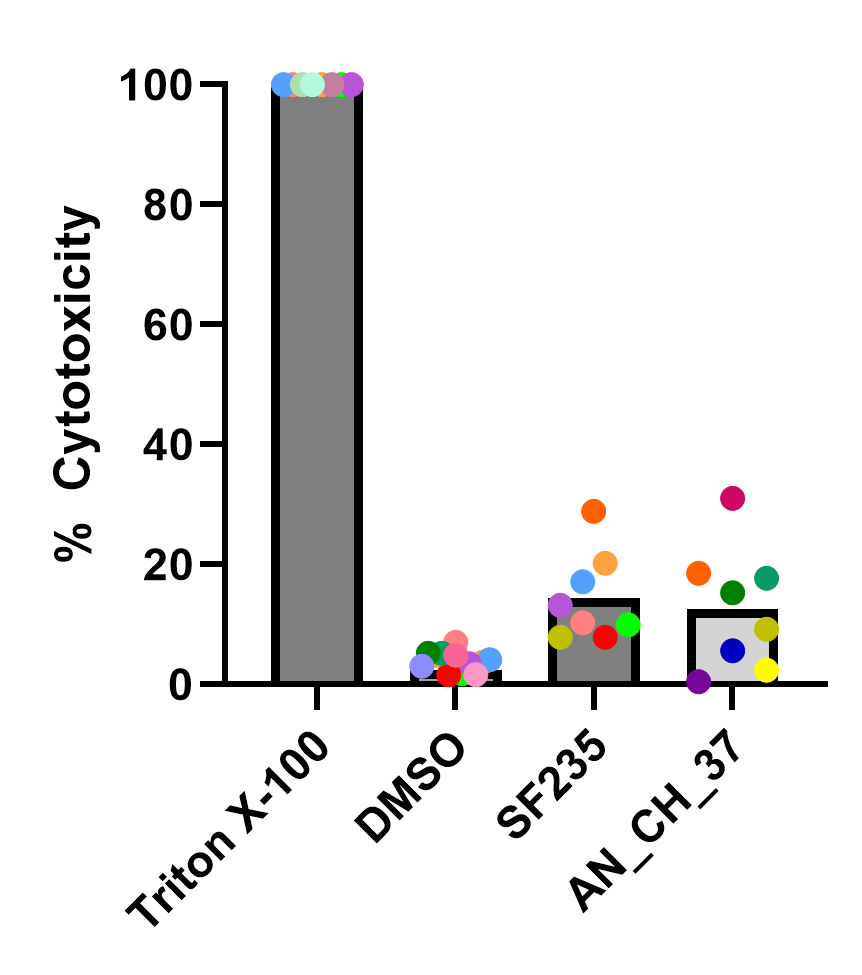

Supplement: Supplementary Figure 2 — Inhibitor-induced cytotoxicity. Cytotoxicity was measured after J774A.1 macrophage cells were incubated with SF235 (50 μM), AN_CH_37 (50 μM) or control (1% DMSO) for 24 hours using Roche LDH Cell Cytotoxicity kit. n=8 for SF235 and AN_CH_37; n=16 for DMSO. [file Image_2.tif]

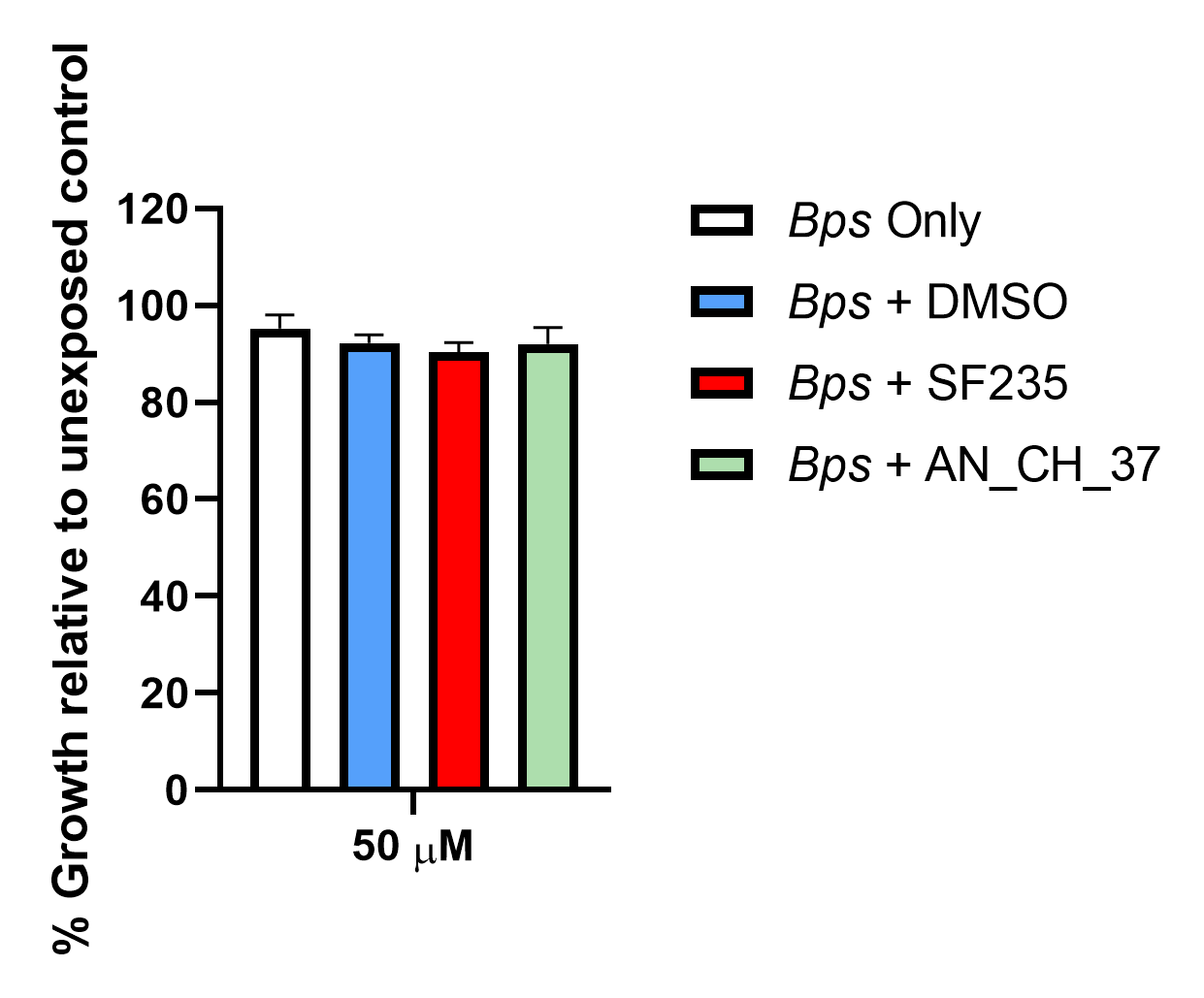

Supplement: Supplementary Figure 3 — Growth kinetics of B. pseudomallei in the presence and absence of Mip inhibitor SF235, AN_CH_37 and vehicle control (DMSO) in Luria Broth. Values are the mean MICs from three biological replicates. MIC was determined by measuring the optical density at 590 nm at 24 hours after DMSO or inhibitor exposure, and the MIC was called the lowest concentration that resulted in less than 20% growth of the unexposed control on that plate. n=8 for SF235 and AN_CH_37; n=16 for DMSO. [file Image_3.tif]
